# Applying system dynamics methods for local obesity prevention: results from a group model building study in Amsterdam, the Netherlands

**DOI:** 10.1186/s12961-025-01398-6

**Published:** 2025-10-14

**Authors:** Wilma Waterlander, Angie Luna Pinzon, Coosje Dijkstra, Teatske Altenburg, Meredith L. Overman, Manou Anselma, Naomi de Pooter, Vincent Busch, Mai Chinapaw, Karien Stronks

**Affiliations:** 1 Amsterdam UMC, Department of Public and Occupational Health, Location University of Amsterdam, Amsterdam Public Health Research Institute, Amsterdam, the Netherlands; 2 Amsterdam UMC, Location Vrije Universiteit, Department of Public and Occupational Health, Amsterdam Public Health Research Institute, Amsterdam, the Netherlands; 3 Department of Health Promotion, NUTRIM School of Nutrition and Translational Research in Metabolism, Maastricht University, 6229ER Maastricht, The Netherlands; 4 Mulier Institute, Utrecht, the Netherlands; 5 Utrecht University, Utrecht, the Netherlands; 6 Sarphati Amsterdam, Public Health Service (GGD), City of Amsterdam, Nieuwe Achtergracht 100, 1018WT Amsterdam, The Netherlands

**Keywords:** Group model building, System dynamics, Obesity prevention, Systems thinking, Systems change, Public health, Leverage points

## Abstract

**Background:**

This study aimed to evaluate a participatory system dynamics group model building (GMB) process for local obesity prevention and policy.

**Methods:**

GMB workshops with *n* = 9 to *n* = 31 participants were held with local stakeholders involved in child health in a community setting in Amsterdam, the Netherlands. The traditional number of two GMB workshops was expanded to a total of five workshops to facilitate the process of system understanding (workshop 1 and 2) and leverage point and action development (workshops 3–5). Also, four thematic sub-groups were installed to work on systems change. We triangulated different data sources to evaluate the outcomes, including stakeholder interviews at two time points.

**Results:**

The GMB workshops resulted in a casual loop diagram (CLD) with 30 mechanisms explaining the obesity problem, five potential leverage points for change and 16 action ideas; which were subsequently classified using the intervention level framework. Action ideas targeted system elements (*n* = 3); feedback and delay (*n* = 4); structure (*n* = 8); and goals (*n* = 1). Interviews revealed the challenge in pushing beyond superficial solutions and instead developing initiatives that can achieve fundamental changes in the underlying system dynamics.

**Conclusions:**

Our study is one of the first providing insight into how system understanding can be translated into actionable leverage points and action ideas targeting different system levels. Achieving tangible systems change at system goals or paradigm level requires substantial stakeholder involvement and a dedicated process.

## Background

There is growing support for the value of participatory methods based in system dynamics in childhood obesity prevention. One method that is growing in popularity is group model building (GMB). GMB is a participatory qualitative systems method aimed at engaging various stakeholders to collectively consider the causes of complex problems [1]. GMB does not follow a uniform approach but consists of a variety of practical exercises (or “scripts”) that can be combined and tailored to fit different workshop formats [1]. Through this method, a detailed understanding of the targeted system is captured in causal loop diagrams (CLD), visualizing the factors underlying the problem, the connections between these factors and feedback loops. The method has been applied in many different fields outside public health, including business and organizational change, sustainability research and education [2].

Examples of GMB studies on childhood obesity prevention include a study by Savona et al. [3] whereby *n* = 257 adolescents identified interrelated drivers of obesity, including: commercial drivers of unhealthy diets; mental health and unhealthy diets; social media use, body image and motivation to exercise [3]. Also, Brennan et al. conducted GMB workshops to study healthy living in relation to childhood obesity and identified five sub-systems, including: healthy eating policies and environments; active living policies and environments; health and health behaviours; partnership and community capacity; and social determinants [4]. GMB has also shown promise for application in diverse communities; for example, Browne et al. showed that GMB is a promising tool for bringing together Aboriginal and non-Aboriginal knowledge, which Aboriginal communities could utilize to explore and address complex problems in a manner that is consistent with their worldviews [5].

According to systems theory, understanding a system can serve as “input” for systems changes, through the identification of leverage points [6]. While, overall, GMB has been shown to be helpful in gaining system understating through engaging groups of stakeholders [7] and generating collective mental models [8], the degree to which it can enact congruent systems change is less clear [2, 9]. Most studies report the constructed CLD as primary outcome without further systems analysis or intervention. One example showing how GMB can ignite systems change comes from a study by Allender et al. [10] revealing how their system dynamics approach helped building new relationships and helped identifying system leverage points including: willingness to take risks; and redesigning health promotion work to have a community development focus [10]. However, the authors also concluded that concrete actions targeting the system as a whole were minimal. They suggested that further empirical studies are needed to deepen the understanding of the elements of success in systems approaches, in particular in relation to whether system understanding can lead to systems change to prevent childhood obesity [10]. Similarly, Ryom et al. reported challenges regarding the usefulness of the system mapping process and how to bridge the obtained deeper understanding of the complex system of childhood overweight and obesity into concrete ideas to change the system [11].

The aim of this study was to evaluate a participatory system dynamics group model building (GMB) process for local obesity prevention and policy with a focus on the process between system understanding and systems change in Amsterdam, the Netherlands.

## Methods

### LIKE programme

This study was part of the larger Lifestyle Innovations based on youth Knowledge and Experience (LIKE) programme. LIKE is embedded in the Amsterdam Healthy Weight Programme (AHWP), which is a local-government-led whole systems approach with the long-term goal of reducing childhood overweight and obesity [12]. LIKE specifically focuses on the transition from child to adolescent (age 10–14 years) and uses a system dynamics and participatory action research approach [13]. This study was part of the first two stages of the LIKE programme, aiming to map the pre-existing system of obesity-related behaviours [14, 15].

### Design

We conducted five rounds of GMB workshops between January 2020 and June 2021 in a community setting in Amsterdam, and included follow-up data until October 2022. The first two GMB workshops focused on gaining system understanding and the final three workshops (online) on identifying leverage points. We had to switch to an online format owing to COVID-19 measures. The GMB workshops resulted in a CLD, leverage points, action ideas and action plans; which were subsequently classified by the LIKE project team according to the targeted systems level using the intervention level framework (ILF) [16]. Observations, meeting notes, questionnaires and stakeholder interviews at *t* = 1 (after the third workshop) and *t* = 2 (about 1 year after the final workshop) were used to gain insight into participant perspectives regarding the GMB process.

### Group model building procedures

#### Participants

**Fig. 1:**
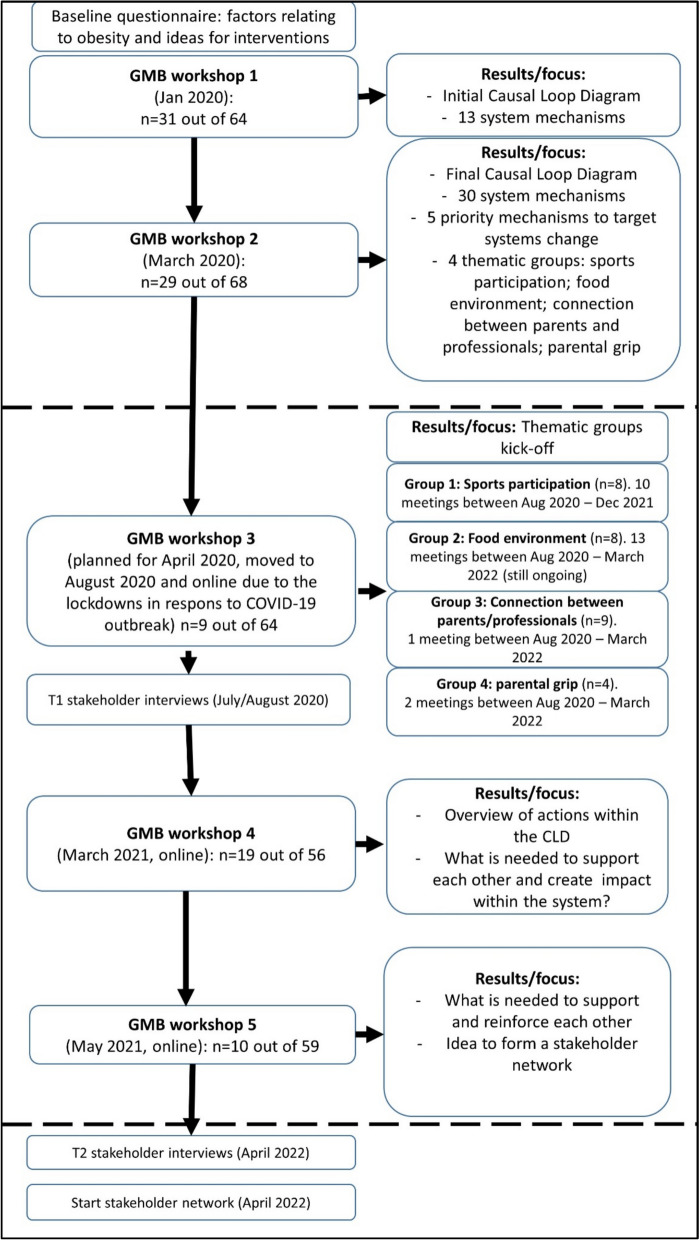
Timeline GMB workshops and thematic sub-groups

Our goal was to select stakeholders who were involved in child health-related activities in Amsterdam and represented the following sectors: local municipality; AHWP; sports clubs; retail; community organizations; schools and youth health care. We primarily chose stakeholders with direct involvement with adolescents, as opposed to those in managerial roles, with the intention of gaining insights into adolescents’ day-to-day living environment. The participants representing community organizations included volunteers, who also participated in their role as parents. Adolescents themselves were not included in the GMB process as they were included in a separate participatory part of the LIKE project [17]. For the first GMB workshop, *n* = 64 stakeholders were invited via email signed by the programme manager of the AHWP. For each subsequent workshop, this list of invitees was updated (Fig. 1).

#### GMB workshops 1 and 2

The first two GMB workshops were organized following the conventions of GMB practice, including four scripts: “hopes and fears”; “graphs over time”; “connection circles” and construction and refinement of the CLD [1]. Each workshop lasted approximately 3.5 h. The central question was: “Which factors influence and/or are influenced by an unhealthy lifestyle in relation to overweight amongst adolescents aged 10–14 years in Amsterdam East?” Within the “hopes and fears” script, participants were asked to express their expectations and concerns regarding the workshop. Next, using the “graphs over time” script, participants were asked to identify as many variables as possible relating to the central question. In sub-groups, participants then came up with a top 5. These top 5 variables were collected plenary, using a wall-builder and connection circle in STICKE software. Participants were then asked to find connections between the variables and their polarity (that is, negative or positive connection). The first GMB workshop ended with a first draft of the CLD.

The first workshop was opened and closed by the convenor, who was the manager of the AHWP. The GMB workshops were otherwise facilitated by a team of researchers who had received training in GMB, including one main facilitator, one modeller, one reflector and four to six sub-group facilitators and/or note-takers. Connection circles and CLDs were developed using STICKE-2 software, developed by Deakin University [18]. During each session, the facilitating team took written notes and reflected before and immediately after the GMB sessions.

In between the first and second workshop, three members of the facilitating team refined the CLD, including removing duplicate variables and adding missing connections based on what participants had expressed during the workshop. Also, this team identified feedback loops and sub-systems which were used to organize the CLD according to the underlying mechanisms. Mechanisms generally included a combination of feedback loops, and they provided a qualitative description of what was observed in the CLD.

For the second workshop, the refined CLD was printed and presented to the participants. The workshop started with a networking exercise, asking participants to place a sticker in the CLD in the area which they thought was most important and/or where they were currently working on. Subsequently, the facilitating team presented the identified mechanisms and corresponding feedback loops, and asked participants whether certain mechanisms were missing. Next, participants identified and ranked the most important mechanisms (rank 1 = 3 points; rank 2 = 2 points; rank 3 = 1 point). We purposively focused on mechanisms rather than isolated variables, to facilitate systems thinking among participants by articulating a narrative of the dynamics depicted in the CLD, and thereby avoid participants falling back into thinking in single determinants. The ranked mechanisms served as a starting point for the subsequent formulation of leverage points, whereby the facilitation team re-framed the mechanisms in terms of a theory of change.

Participants could then choose a leverage point to start working on action idea development for systems change. First, they were given no boundaries and were encouraged to develop “big ideas”. To support this process, participants were encouraged to come up with ideas within the school, community, health care, city or national level. Next, participants were challenged to incorporate wider environmental challenges based on the Analysis Grid for Elements Linked to Obesity (ANGELO) Framework, including regarding the physical, economic, social and political environment [19]. Finally, to make a first step into concrete action plans, each group was asked to fill in a template action form, including who should do what and when. Thematic sub-groups were encouraged to continue working on those ideas prior to the next workshop.

#### GMB workshops 3–5

Often, the GMB process concludes after two workshops; but, to allow for a more structured progression from system understanding to the development of actionable ideas for change, we developed an additional phase. This phase encompassed three more plenary GMB sessions and thematic sub-groups (see “Results” section). GMB workshops 3–5 focused on further refinement of the leverage points and writing action plans with the ultimate goal of actual action implementation. We had small seeding grants available (up to €3000) to support the implementation of ideas. Groups were encouraged to progress their ideas between the workshops. We planned the third GMB workshop round within a month after the second GMB workshop, but had to switch to an online format owing to the outbreak of COVID-19, whereby we experienced a significant delay (6 months between the second and third meeting). To suit the online format, the third workshop round consisted of separate workshops for each of the four thematic sub-groups. In the fourth plenary workshop, we provided an overview of the action ideas thus far and where these were placed in the CLD. We used break-out rooms (in ZOOM) and Miro boards to collect action ideas. The fifth and final meeting focused on supporting collective impact across all thematic sub-groups.

### Data collection

#### Causal loop diagram

The CLD resulting from the first two GMB workshops was transferred to Kumu and was further analysed by two members of the facilitation team, whereby it was checked regarding: missing variables and connections; inconsequent labelling of variables; and missing feedback loops. We used Kumu software in this analysis stage because this software had the option to automatically detect feedback loops.

#### Questionnaires

To examine how participants’ perception of the problem (that is, childhood obesity) changed during the GMB process, participants were asked to fill in a baseline questionnaire asking what they considered to be “the most important factors and solutions relating to overweight and obesity”, prior to the first workshop, which we compared with the CLD and action ideas developed during the GMB process.

#### Observations

Notes and observations were taken by four to six note-takers during all GMB workshops. A topic list was constructed for the note-takers and included questions relating to engagement and levels of tension and/or enthusiasm amongst participants.

#### Interviews

To gain insight into participant perspectives on the GMB process, we conducted interviews with *n* = 4 stakeholders at *t* = 1 (prior to the third GMB workshop) and *n* = 4 stakeholders at *t* = 2 (1 year after the last GMB workshop). For this, we purposively selected participants who had engaged in multiple GMB workshops and selected at least one member of each of the sub-groups. We interviewed different participants at both time points. Interviews were transcribed verbatim, and deductive thematic analysis in Microsoft Word was conducted by two academic researchers. The interview guide was based on a previous GMB study [10] and potential building blocks for building a system thinking prevention workforce [20–22] and previous work relating to the use of micro-grants as a stimulus for community action [23] (see Appendix 1).

## Results

### Gained system understanding

**Fig. 2:**
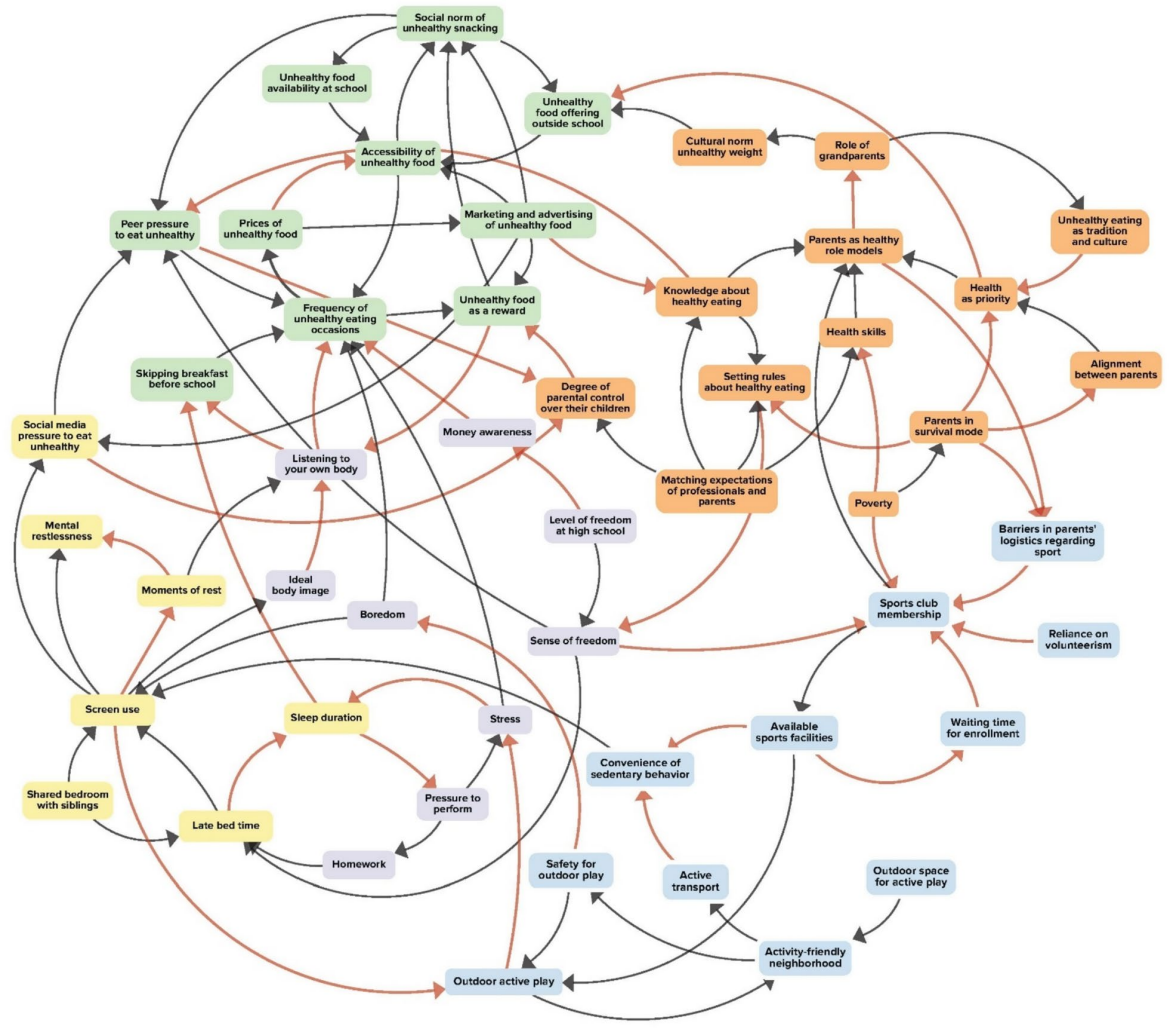
Causal loop diagram. Orange arrows represent positive polarity (more A leads to more B; or less A leads to less B); Black arrows represent negative polarity (more A leads to less B; or less A leads to more B). We identified five sub-systems: “transition to adolescence” (purple); “food environment” (green); “home environment” (orange); “sleep environment” (yellow); and “physical activity environment” (blue)

The first two GMB workshops resulted in a CLD containing 50 factors within five sub-systems, including: food environment; sleep environment; home environment; physical activity opportunities; and transition to adolescence (Fig. 2). Many factors that were listed in the CLD came up during the workshop and were not named by the participants in the baseline questionnaire, such as factors relating to sleep health, pressure to perform, and relationship between parents and health care professionals.

After the first GMB workshop, the LIKE project team identified 17 mechanisms, and during the second workshop participants identified another 13 mechanisms, resulting in a total of 30 mechanisms (Table 1). For example, the first three mechanisms related to a perceived absence of boredom among adolescents; that is, the moment boredom sets in, adolescents tend to reach for their phones. Consequently, genuine moments of rest become scarce, impeding the ability to tune in to one’s own body signals regarding hunger and satiety. This is also connected with the promotion of an idealized body image and the marketing of unhealthy food, exacerbated by peer pressure via social media.

**Table 1:** Factors, mechanisms and leverage points in the CLD

Sub-system		Factors and mechanisms	Identified leverage point
Transition to adolescence		1. (Perceived) **higher** academic pressure and focus on academic skills leads to **less** active play or sports 2. **Less** room for boredom; leads to **more** time on smart phones; leads to **less** real moments of rest. This leads to−> 3. Being **less** able to listening to one’s own body 4. A **more** idealized body image (through social media) also leads to being **less** able to listening one’s own body 5. Adolescents receive **more** freedom from parents and teachers and therefore they also feel they can 6. Claim **more** freedom and own responsibility 7. Relatedly, they have **more** access to their own money, and **less** financial knowledge, whereby they spend **more** money on unhealthy food	**(L5): Role of parents from mentor to coach:** As children transition into adolescence, the role of parents changes from mentor to coach, whereby parents feel that they lack skills to deal with this transition, in particular in relation to technology. Simultaneously parents face multiple responsibilities, meaning that healthy lifestyle choices are not always prioritized
Food environment		8. A **higher** unhealthy food availability leads to a **higher** unhealthy food accessibility, including inside and around school 9. **Lower** prices of unhealthy food make it **more** accessible and **higher** sales of these products makes them **more** profitable and therefore prices can get **lower**, whereby they become again **more** accessible 10. A **higher** accessibility of unhealthy food; makes it **more** normalized; and this norm becomes **stronger** through marketing 11. A **stronger** norm leads to **more** peer pressure to buy unhealthy food; this leads to feelings of hunger or satiety become **less** prominent and also parents have **less** grip on unhealthy food behaviour	**(L3): Restoring the imbalance between healthy and unhealthy food:** there is an imbalance between the availability and power of unhealthy, often internationally branded food retail compared with more healthy local food outlets who often have lower profit margins. From the viewpoint of healthy food availability, local entrepreneurship and social neighbourhoods, it important to create more space for healthy food retail
Home environment		12. Parents have **less** control during the transition to adolescence 13. **More** responsibilities of parents leads to **less** coordination between parents and health being **less** of a priority 14. **Less** solid trust-based connection between parents and professionals 15. With more parents working, the role of grandparents is getting **more** important, in terms of care responsibilities and cultural norms	**(L1): Connect different stakeholders working on healthy youth in Amsterdam:** stakeholders are not very well connected; this is the first time that stakeholders in this community were meeting each other **(L4): Reduce the cultural and communication barriers between parents and health care professionals:** There are cultural and communication barriers between parents and professionals which lead to misunderstandings, which in turn lead to disengagement from both sides
Physical activity opportunities		16. **More** attractive alternatives compared with active transport lead to **less** active transport 17. This leads to neighbourhoods being perceived as **less** safe for active transport 18. **More** urbanization leads to **less** outdoor which leads to **less** outdoor active play. This leads to 19. **More** screen time and again **more** perceptions of unsafety for outdoor play 20. **More** waiting lists for younger children, leads to **less** sport club memberships 21. This leads to a **lower** availability of sports clubs nearby and 22. **Less** opportunities to combine after-school care with sports participation which leads to 23. **More** complicated logistics for parents to take their children to sports activities and 24. Sports involvement requires **more** of parents’ voluntary contribution of time 25. Parents serve **less** often as a role model regarding sports	**(L2): Focus on the transition from child to teenager** during which children become less involved in organized sports, particularly girls;
Sleep environment		26. **More** social pressure for screen use leads to 27. **More** screen use 28. **More** screen use leads to **less** moments of rest 29. **More** shared bedrooms lead to 30. **More** mental unrest and **more** sleepiness which again leads to **more** screen use and a **higher** frequency of unhealthy eating,	Not identified

Bold text indicates the polarity between factors

Participants prioritized the 30 mechanisms; resulting in a top 5. These mechanisms were then reworded into leverage points, and included: (1) connecting stakeholders working on healthy youth in Amsterdam; (2) focus on the transition from child to teenager where, particularly girls, become less involved in organized sports; (3) the imbalance between the availability of unhealthy, often internationally branded food retail, and more healthy local food outlets; (4) the cultural distance between parents and healthcare professionals; and (5) the changing role of parents during adolescence (Table 1).

### Systems action: results from the thematic sub-groups

Following the first two GMB workshops, participants formed four thematic sub-groups around the identified leverage points. Each group organized their own meetings in addition to the five plenary GMB workshops, and the number of meetings varied from 1 (“connection between parents and healthcare professionals” group) to 13 meetings (“food environment” group) (Fig. 1).

**Fig. 3:**
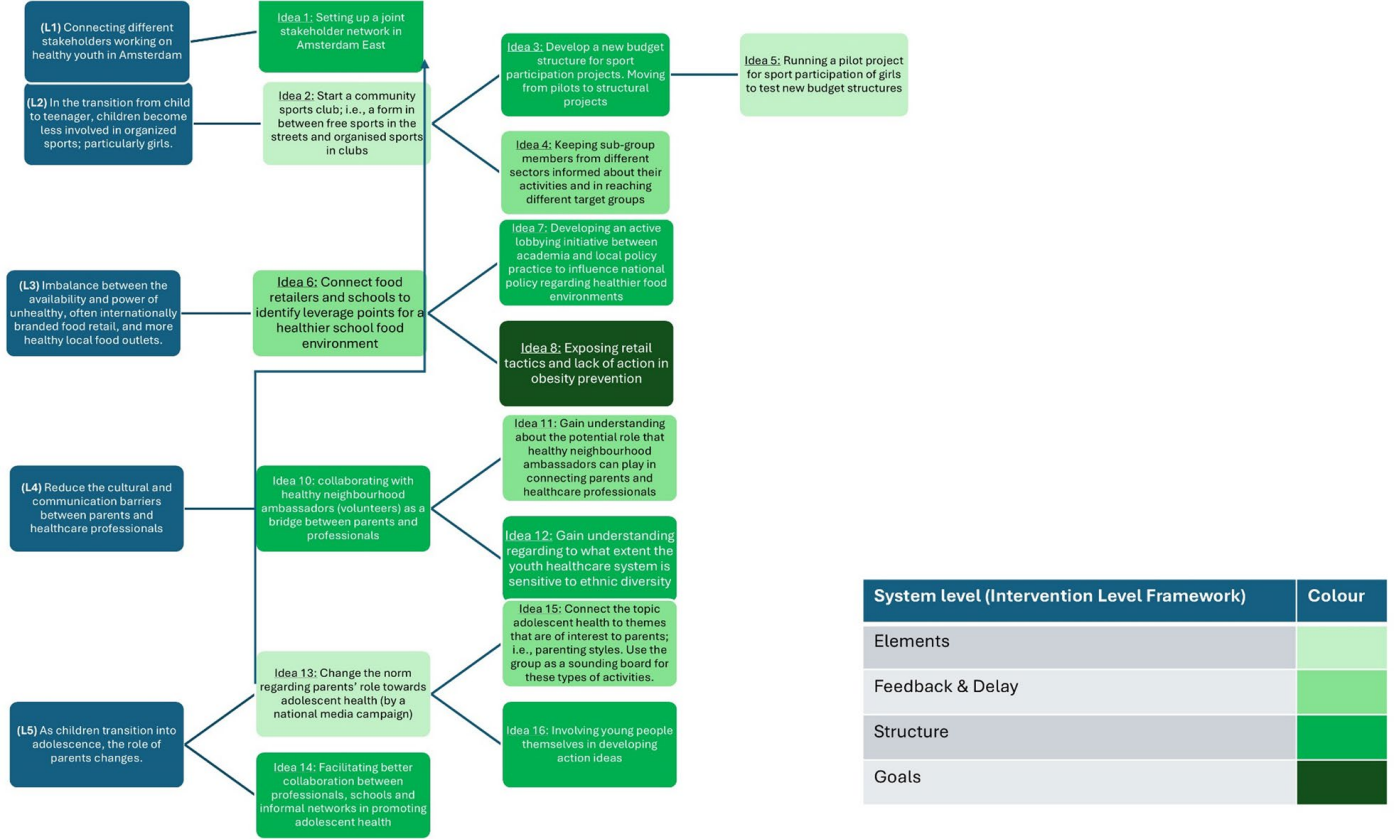
Overview of leverage points and formed action ideas, targeted system level and adaptation

Prior to the GMB workshops, suggested action ideas were mainly focused on the level of system elements such as stricter school policies or educating parents. During the GMB process, sub-groups came up with 16 action ideas targeting different system levels, including: elements (*n* = 3); feedback and delay (*n* = 4); system structure (*n* = 8); and system goals (*n* = 1) (Fig. 3). Most of the ideas adapted over time. For example, the “sports participation” sub-group’s initial idea was to install a community sports club. Subsequently, they learned that it would be more valuable to work on budget structures for sports projects because this would bring more long-term security to already ongoing initiatives. Thereby, the idea evolved from targeting system level 1 (system elements) to system level 3 (system structure). However, before this idea could be implemented, the group first developed a pilot project to gain understanding on how new budget structures should be organized (back to system level 1, elements). The “food environment” sub-group did not go ahead after the second GMB workshop. Therefore, the LIKE project team formed a new sub-group and invited other relevant participants, mostly from the municipality. The idea evolved from working with supermarkets and schools in the community to make the food environment healthier (system level 2), to examining the underlying mechanisms as to why supermarkets were reluctant in making meaningful progress in supplying more healthy food (system level 4).

Most actions halted during the process of discussing and refining the idea with stakeholders within the given context. Some of the ideas were adopted by the involved stakeholders, for example, within their daily jobs. Other ideas stranded because none of the group members had sufficient time to lead the idea. Therefore, we ignited a separate process within the broader LIKE project where we worked with colleagues involved in the LIKE consortium to advance ideas beyond the scope of the GMB process. Details about how actions changed over time and the value of the GMB process are provided below.

### Reflections on the GMB process

The number of participants that attended the GMB workshops was *n*_1_ = 31; *n*_2_ = 29; *n*_3_ = 9; *n*_4_ = 19; and *n*_5_ = 10, respectively (Fig. 1 and Appendix 2). At *t* = 1 interviewees represented the sectors “local municipality” (*n* = 1), “civil society organizations” (*n* = 2) and “local retail” (*n* = 1). At *t* = 2, this was “local municipality” (*n* = 2, different levels of the organization) and “the Amsterdam Healthy Weight Programme” (*n* = 2).

Below, we report some key reflections following the GMB workshops where we consolidated the primary findings from interviews and facilitators’ reflections into four main themes.

*Theme 1: Participants’ experience during the GMB workshops*

Participants in particular valued the GMB networking element, where multiple participants commented that, although they all work on the same topic in the same community, they had never been in one room all together. On the basis of these findings, the local municipality initiated a new network, with a first meeting in April 2022. Relatedly, participants explained that they valued how the diverse group of stakeholders generated a broader systems view. Nevertheless, we also observed that this divergence of expertise made it challenging for everyone to stay engaged. The plenary GMB meetings focused on providing an overview of all the ongoing initiatives and served as a way to maintain the systems perspective. However, some participants did not attend these meetings because the general character conflicted with other responsibilities more closely aligned to their specific working area. Although they recognized the value of sharing their progress to the wider group and using feedback to take an idea to a next level, it was difficult to prioritize.

*Theme 2: Systems thinking concepts*

Observation records and interview results both revealed that participants valued the GMB workshops in gaining a complete picture of the system and who is doing what in the community. Furthermore, we observed that participants were able to identify mechanisms and discuss systems structures during the GMB workshops. One interview participant noted:

“I knew about all the sport-related activities already, but this really encouraged me to look for the combination with things like parenting styles, problems in the home setting, healthy food, involvement of parents..” [R2, Amsterdam East Municipality].

“..I can’t really give a concrete example, but it turned on a light in my head... we can come up with all these nice sport activities, but when clubs have no room for these ideas, there is no point in doing this..”[R2, Amsterdam East Municipality].

*Theme 3: Developing action ideas at different system levels*

Participants were able to formulate leverage points and come up with action ideas at different system levels and were able to formulate concrete action plans. Also, the process encouraged participants to adapt their ideas on the basis of new insights. However, implementation of these ideas required investment outside, or in addition to, the GMB process. This additional investment also revealed a specific tension as it required both applying the learnings in their regular job and continuing to attend the GMB sessions. Similarly, participants reported that it was difficult to work around existing system structures to progress their ideas:

“It is also the structure of hourly invoices. We like to work more integral and for example combine mental and physical health, but in the end, the dietician will say, how can I justify that, how can I justify those hours?” [R4, Amsterdam Healthy Weight Approach].

“Yes, these problems already existed, and we with our little action group can make no exceptions, that has to be decided at city level and requires freeing up millions... “[R2, Amsterdam East district].

When asked who else would be needed to progress ideas into actual implementation, it was noted that you need both the people working on the ground, to understand the details, and people in leading position who have oversight and the ability to influence leverage points at deeper system levels. The majority of the action ideas remained in the conceptual stage and the action ideas targeting deeper system levels (goals or paradigm) encountered resistance and were often scaled down into more manageable ideas. This reveals the challenge in pushing beyond superficial solutions and instead developing and implementing initiatives that aim to bring about fundamental changes in the underlying system dynamics. This was also mentioned in one of the interviews:

“However, a challenge arises because the professionals involved in the GMB process seek prompt, tangible results. This creates a clash, between the need for immediate outcomes and the more time-consuming process of systems change.” [R2 Amsterdam East district].

## Discussion

The aim of this study was to evaluate the results of a GMB process on gaining system understanding and as a starting point for system changes for local obesity prevention. Earlier studies highlight the need for further in‐depth empirical studies on the process of GMB and how gained system understanding can facilitate systems change [10, 24].

Our findings show how the GMB process was valuable in gaining system understanding by generating a comprehensive CLD and by activating systems thinking amongst participants. Visualizing the complexity of the problem in the form of a CLD helped participants understand their own position in the system and the need for more congruent action. Also, our study reveals how this system understanding could be used as a starting point for systems action by identifying leverage points and generating action ideas targeting different system levels. Action ideas adapted over time on the basis of growing insights in the system and the context in which actions were to be implemented. For example, action ideas were abandoned when participants learned that such actions would not provide a sustainable solution, thereby shifting focus from systems elements (for example, starting a new initiative) to systems structure (for example, changing subsidy structures). Nevertheless, our findings also reveal the challenge in navigating change at deeper system levels, because this requires zooming in and out of the system and a parallel process of involving a continuously changing mix of stakeholders. The development and implementation of actions targeting deeper system levels is generally underrepresented in the published obesity prevention literature to date [25].

Our finding that systems mapping in the form of CLDs is useful in gaining system understanding is supported by other studies [9]. Brennan et al. reported that, through the construction of CLDs, people can share mental models which helps in improving thinking about the structure of a problem as well as recognizing more immediate versus delayed responses, unintended side effects and sources of policy resistance [4]. Similar to Brennan et al., we found that the GMB process supported the development of a common understanding of the system across stakeholders [4]. We found at baseline that, individually, participants came up with two to maximum five determinants of obesity, while collectively, they were able to identify 30 mechanisms and five leverage points to be targeted for systems change. These mechanisms and leverage points were substantially different from the originally named determinants at baseline.

A related finding is that, prior to the GMB workshops, in the baseline questionnaire, suggested action ideas focused mainly on system elements; while during the GMB process, a much wider range of ideas were generated, targeting different systems levels. However, our study also highlights that understanding the level of complexity does not automatically translate into implemented actions that can facilitate tangible systems change: a phenomenon called the “abstraction gap” where people can only think of strategies that are in their own heads [26]. This was also observed in earlier studies. For example, a recent Danish study by Ryom et al. illustrates how system mapping can provoke tensions − specifically, between viewing the map as a practical step toward action and as a conceptual tool for unpacking the complexity of the problem [11]. Furthermore, a paper by Gerritsen et al. [27] revealed that a GMB process was successful at shifting participants’ thinking towards understanding the systemic barriers, but that it was much more difficult for participants to identify actions that could potentially influence the deeper systemic issues relating to culture, social norms and the underlying goals of the system [27]. Therefore, in our study, we installed a dedicated process for this purpose with specific attention to the development of action ideas at different system levels and allowing a time span of more than 2 years, in a context where already a lot was happening owing to the existence of the AHWP. Nevertheless, it remained difficult to achieve tangible change at deeper systems levels. This underscores the inherent complexity of the problem of obesity that we studied. Adopting a system dynamics approach recognizes this complexity, but a GMB process with local stakeholders alone cannot instantly resolve this. It does show the level of effort that is needed to achieve systems change, and it provides points of references for improving the process. Brown et al. developed a theory of change in the form of a stock-and-flow diagram showing important elements that are required in community-based system interventions, including: research support; community involvement, collaboration, quality of action, feedback about intervention success, and how leadership interacts with community health behaviours and outcomes [28]. Some of these elements were well represented in our study, such as research support, community involvement and collaboration; but quality of actions, feedback and leadership were less well developed.

A recent review identified only three studies that comprehensively applied a system dynamics approach to obesity prevention in intervention development [29]. Our study provides valuable insights into what actions based on principles of system dynamics look like [30]. For instance, comprehending the reasons behind the lack of sustainability in specific sports projects necessitated a detailed understanding of funding structures and an analysis of the needs of the target group (young girls), along with an exploration of why these components failed to align. When attempting to tackle these issues, the sub-group found themselves at a loss on how to proceed. They sought inspiration from other groups or stakeholders operating in different facets of the system, yet they fell short of reaching the crucial stage of “escalating back” towards those people in the system that could actually change things, that is, those in managerial roles. This finding reveals the need and complexity of involving all key stakeholders in different stages of the process, as well as a team of experts in system dynamics [31]. In future projects, this capacity for people to identify their role in complex adaptive systems and their ability to influence others and advocating for change is of key interest [22]. A recent example of such an approach is a study by Heemskerk et al. that developed a whole-systems action plan based on an earlier CLD, providing insights into which leverage points to target and what actions would fit. However, the resulting action plan has not yet been implemented, so it is unclear how that would work in practice [32].

A related finding pertains to the concept that our understanding of what constitutes an action or intervention must be reconsidered when conducting a study within a system dynamics approach. Traditionally, we are inclined to think of a tangible intervention, such as implementing a healthier canteen. However, within this GMB process, we observed that action development occurred in a different manner. Participants became aware of the complexity, and generated ideas that would intervene at deeper layers of the system through the use of leverage points. Subsequently, they engaged with these ideas, often discovering that it prompted further questions. This evolution altered ideas over time and initiated new concepts. While this method of action development may not immediately lead to concrete new implemented interventions, as evident in our study, it does set certain processes in motion. We described this process, as part of the overall LIKE project, in more detail in a separate paper [33]. For example, an earlier study revealed the mechanisms via which community-based system dynamics can work to facilitate system change by developing shared language and ownership among stakeholders [34]. An intervention is then an event in the history of a system, leading to the evolution of new structures and new shared meanings and redistributing and transforming resources [35]. Our study gives an idea of what this could look like.

### Strengths and limitations

A strength of our study is that we applied a comprehensive GMB approach consisting of plenary workshops and thematic sub-groups which were followed for more than 2 years [36]. We did this to get a grip of what is needed to translate the gained system understanding, usually generated after two to three GMB workshops, into systems change. Another strength is that we collaborated with the AHWP and therefore had a strong community partner in developing and implementing the GMB process. However, a limitation was that we did not explicitly discuss ownership of the GMB process. For future studies, we recommend to explicitly discuss ownership, and how everyone envisions the involvement of relevant stakeholders and their responsibilities [37]. Another limitation is that part of the process happened online owing to COVID-19 restrictions. While this could also be seen as a strength because it facilitated meetings between people working in different locations, it happened at a crucial stage in the GMB process (in moving from understanding the system to action) and caused significant delays. The online meetings also led to disengagement from some of the participants [38].

## Conclusions

Our study shows how GMB can be valuable in generating systems change in a local context. Visualizing the complexity of the problem in the form of a CLD helped participants understand their position in the system and the need for more congruent action. Identifying leverage points and allowing action ideas to adapt over time supported generating action ideas targeting different system levels. However, instigating tangible systems change is a distinct challenge, necessitating the implementation of a dedicated process and acknowledging the complexity of and long-term effort required for the desired transformation. A clear delineation of roles and responsibilities among involved stakeholders, coupled with a flexible approach attuned to contextual and systemic needs, is required to support this process.

## Data Availability

No datasets were generated or analysed during the current study.

## Appendix 1. Overview of the interview questions

**Table:**

	Theme	Items
*t* = 1 (prior to the third GMB workshop)	Participants’ expectations and general experiences during the first two GMB workshops	− How did the workshops influence participants thinking about the problem and/or potential leverage points? − What were the main learnings from the workshops relating to systems thinking? − How did the workshops influence participants’ network and relationships? − What would participants like to do for the health of adolescents if they were the mayor of Amsterdam?
*t* = 2 (1 year after the final GMB workshop)	Value of the entire GMB process	– Participants’ general impression of the GMB process; the value of building a CLD; – Whether participants had changed their practices compared with the start of 2020; – What participants believed would be the best way to engage stakeholders in future projects; – How to move from system understanding to systems change
	Process of generating action ideas	– What participants believed were important barriers and facilitators for developing actions within the system and what factors would enable/hinder them to target different system levels; – The quality of the facilitation of the thematic sub-groups; – Whether action ideas should be developed bottom up or top down; – What they thought is needed next to achieve systems change
	How to build a system thinking prevention system and workforce and stimulate community action for systems change	– What is needed in terms of leadership; – How to provide (real time) feedback on data and progress; – Available resources for systems change (including infrastructure and organizational capacity); – The role of relationships

## Appendix 2: Overview of participants per sector for the five GMB workshops

**Table:**

		Workshop 1: CLD building	Workshop 2: mechanisms and leverage points	Workshop 3: action ideas	Workshop 4: collaboration between sub-groups	Workshop 5: impact from the group as a whole
Participants per sector	Total	*N* = 31	*N* = 29	*N* = 9	*N* = 19	*N* = 10
	Local municipality	6	8	3	7	2
	Amsterdam Healthy Weight Programme/Public Health Service Amsterdam	10	7	3	4	2
	Sports clubs	3	3	3	1	2
	Local retail	2	1	-	1	-
	Civil society organizations	4	5	1	3	2
	Schools	3	-	-	-	-
	Youth health care	3	3	-	3	1
